# Metformin-Induced Apoptosis Is Mediated Through Mitochondrial VDAC1

**DOI:** 10.3390/ph18050757

**Published:** 2025-05-20

**Authors:** Anna Shteinfer-Kuzmine, Meital M. Moyal, Aditya Karunanithi Nivedita, Sweta Trishna, Almog Nadir, Shubhandra Tripathi, Varda Shoshan-Barmatz

**Affiliations:** 1 National Institute for Biotechnology in the Negev, Ben-Gurion University of the Negev, Beer-Sheva 84105, Israel; shteinfe@post.bgu.ac.il; 2 Department of Life Sciences, Ben-Gurion University of the Negev, Beer-Sheva 84105, Israel; petrescu@post.bgu.ac.il (M.M.M.); nivedita@post.bgu.ac.il (A.K.N.); trishna@post.bgu.ac.il (S.T.); nadiral@post.bgu.ac.il (A.N.); 3 Department of Chemistry, Indian Institute of Technology, Kanpur 208016, India; shub.bioinfo@gmail.com

**Keywords:** apoptosis, hexokinase, metformin, mitochondria, VDAC1

## Abstract

**Background:** Besides diabetes mellitus, metformin has been identified as a potential therapeutic agent for treating various other conditions that include various cancers, cardiovascular diseases, neurodegenerative diseases, and aging. In cancer, metformin increased apoptotic cell death, while inhibiting it in neurodegenerative diseases. Thus, different modes of metformin action at the molecular level have been proposed. **Methods:** In this study, we present the mitochondria and the VDAC1 (voltage-dependent anion channel) as a potential target of metformin. **Results:** Metformin induces VDAC1 overexpression, its oligomerization, and subsequent apoptosis. Metformin analogs phenformin and buformin at much lower concentrations also induce VDAC1 overexpression, oligomerization, and cell death. We demonstrate the interaction of metformin with purified VDAC1, which inhibited its channel conduction in a voltage-dependent manner. Metformin bound to the synthetic VDAC1-*N*-terminal peptide and binding to this domain was also found by its molecular docking with VDAC1. Moreover, we demonstrated metformin binding to purified hexokinases (HK-I) with a 400-fold lower metformin concentration than that required for cell death induction. In cells, metformin induced HK-I detachment from the mitochondrial VDAC1. Lastly, metformin increased the expression of NLRP3 and ASC and induced their co-localization, suggesting inflammasome activation. **Conclusions:** The results suggest that VDAC1 is a target for metformin and its analogs, and this is associated with metformin’s adverse effects on many diseases.

## 1. Introduction

Metformin, a biguanide derivative (3-(diaminomethylidene)-1,1-dimethylguanidine), was originally derived from the flowers of goat’s rue, also known as French lilac (Galega officinalis) [1]. Metformin is the primary treatment for type 2 diabetes mellitus (T2DM) and pre-diabetes, as it temporarily prevents or delays the onset of the disease [2,3]. Beyond its application in T2DM, metformin exhibits diverse effects on several other diseases, including cancer and cardiovascular and neurodegenerative diseases [4,5,6,7,8,9,10,11,12]. Additionally, metformin treatment has been linked to various forms of age-related cognitive decline [13], with studies reporting mixed outcomes, including both positive and negative findings [14]. The complex and diverse molecular nature of these diseases indicates that metformin influences a wide range of biological signaling pathways [15,16]. As a result, identifying its precise mechanisms of action remains challenging. It is proposed that metformin influences cellular metabolism, which is closely connected to the signaling pathways regulating proliferation and survival—a processes often dysregulated in various diseases [4].

These signaling pathways include energy sensing through AMP-activated kinase (AMPK) signaling, which reduces the expression of genes involved in gluconeogenesis, the mammalian target of rapamycin (mTOR), and inflammatory, mitochondrial, insulin, and cell death signaling—each of which plays a role in the development of certain diseases when disrupted [4,15,16].

Mitochondrial dysfunction is shown to be affected by metformin and is common in many diseases, including T2DM, cancer, cardiovascular disease, and various neurodegenerative diseases [17,18]. Indeed, one of the proposed metformin targets is the mitochondrial respiratory chain protein complex-I [4,19,20]. Metformin inhibits complex-I (NADH dehydrogenase) activity, leading to reduced ATP production and elevated levels of AMP and ADP. The resulting increase in the AMP/ATP ratio, along with elevated AMP levels, inhibits the gluconeogenesis and activation of AMPK [4]. Efforts to improve the efficacy of metformin have involved structural modifications, such as the attachment of alkyl or aromatic groups (e.g., buformin and phenformin) [21]. Phenformin, a biguanide derivative, was initially used as an anti-diabetic drug, but it has been withdrawn due to its association with lactic acidosis [22]. However, recent studies have shown that phenformin and other biguanides have potential anti-cancer properties [23].

Phenformin is more potent than metformin, primarily due to differences in cellular uptake. Metformin is highly hydrophobic and relies on organic cation transporters (OCTs) to cross the cell membrane [24,25].

Like metformin, phenformin acts by inhibiting mitochondrial complex I and activating the AMPK pathway, thus increasing the cellular AMP/ATP ratio and the subsequent overproduction of reactive oxygen species (ROS) [25]. AMPK activation leads to the inhibition of mTOR, a central regulator of cell growth and proliferation. Phenformin was also found to inhibit cell proliferation and induce G1 cell cycle arrest and apoptosis [26].

Buformin is another member of the biguanide class of drugs, and it is similar to metformin and phenformin. It lowers blood glucose by improving insulin sensitivity and decreasing hepatic gluconeogenesis [27]. Similarly to phenformin, it use as an anti-diabetic drug was withdrawn due to concerns over lactic acidosis. It also exhibits anti-cancer effects, reducing the proliferation of cancer cells through AMPK activation and mTOR inhibition. Some pre-clinical studies have suggested that buformin may be effective against various cancers, including breast and lung cancers [28], as well as pancreatic tumor [29].

Other metformin analogs include the fluorobenzyl metformin derivatives [30] and the sulfonamide derivatives [31]. Recently, metformin analogs targeted to the mitochondria by having an alkyl chain containing a triphenylphosphonium cation (TPP^+^) were found to have strong anti-cancer activities in pancreatic ductal adenocarcinoma—up to 1000-fold higher potency than metformin [32].

Given metformin’s adverse effects on various diseases where voltage-dependent anion channel 1 (VDAC1) is overexpressed, we proposed VDAC1 as a potential target for metformin [33]. VDAC1 is a multi-functional protein that serves as a key regulator of mitochondrial function, controlling the metabolic and energetic crosstalk between the mitochondria and the rest of the cell [34,35]. VDAC1 plays a crucial role in regulating ATP production, Ca^2+^ homeostasis, and the execution of apoptosis—all indispensable for proper mitochondrial function and overall cellular physiology. Additionally, it is a key protein in mitochondria-mediated apoptotic pathways [34,35,36]. VDAC1 is also proposed as a component of the mitochondrial permeability transition pore [37,38]. Therefore, alterations in the functions of VDAC1, which is a mitochondria gatekeeper, are associated with mitochondrial dysfunction and diseases [36].

In mammals, three isoforms of VDAC (VDAC1, VDAC2, and VDAC3) have been identified, each sharing some, but not all, structural and functional properties. The three isoforms are expressed in most tissue types, with VDAC1 typically exhibiting higher expression levels than VDAC2 and VDAC3 in most, but not all, tissues. Moreover, it is the most abundant and best-studied isoform [39]. Structurally, VDAC1 is composed of 19 transmembrane β-strands connected by flexible loops, forming a β-barrel with a 26-residue-long *N*-terminal region lying inside the pore. The *N*-terminus domain, however, can translocate from the internal pore to the channel surface [40], where it interacts with hexokinase (HK) and other proteins such as Bcl-2, Bcl-xL [34,41,42,43,44,45], and Aβ [46]. Purified and membrane-embedded VDAC1 can assemble into dimers, trimers, tetramers, hexamers, and higher-order complexes [40,43,47,48]. Interacting sites between VDAC1 molecules within the dimers and higher-order oligomers have also been identified [49].

In addition to regulating cellular energy and metabolism, VDAC1 plays a critical role in mitochondria-mediated apoptosis, by facilitating the release of apoptotic proteins and interacting with anti-apoptotic proteins such as Bcl-2, Bcl-xL, and HK [34,41,42,43,44,45]. Several hypotheses have been proposed regarding the mechanism of mitochondria-mediated apoptosis [34]. Our studies, along with others, have shown VDAC1 expression levels increase following apoptosis induction by various reagents—including chemotherapy drugs, arbutin, prednisolone, cisplatin, viral proteins, elevated cytosolic Ca^2+^, or UV irradiation [36]. This upregulation triggers VDAC1 oligomerization, forming a large pore that facilitates the release of mitochondrial pro-apoptotic proteins [48,50]. We further demonstrate that VDAC1 oligomerization is a dynamic process [48,50,51,52].

Oligomeric VDAC1 also mediates mitochondria DNA (mtDNA) release, which triggers type-Ι interferon signaling and inflammation [53,54]. VDAC1 oligomerization is required for the assembly and activation of the NLRP3 inflammasome [55]. Recently, we identified VDAC1-interacting molecules such as diphenylamine-2-carboxylate (DPC) [52] and new molecules developed in our lab to inhibit VDAC1 oligomerization and its subsequent apoptosis—VBIT-4 and VBIT-12 [50].

The association of VDAC1 with various diseases is evidenced by its overexpression. VDAC1 is overexpressed in cancer [36,56,57], Alzheimer’s disease (AD) [58,59,60], T2DM [61,62,63], autoimmune diseases such as lupus [53], cardiovascular diseases (CVDs) [64,65,66,67,68,69], inflammatory bowel diseases (IBDs) [70], non-alcoholic fatty liver disease (NAFLD) [71], COVID-19 [72,73], and others. As VDAC1 overexpression induces apoptotic cell death [51], its elevated levels in these diseases may represent a common pathological mechanism [36].

In a recent review [33], we discussed the adverse effects of metformin across a range of conditions, including cancer, cardiovascular diseases, diabetic kidney disease, neurodegenerative diseases, renal diseases, obesity, inflammation, COVID-19 in diabetic patients, and aging. Our focus was on mitochondrial dysfunction, particularly their role in energy metabolism and cell death, and we raised the question of how a single drug could have seemingly opposing effects, such as promoting apoptotic cell death in cancer while inhibiting it in neurodegenerative diseases. Given that VDAC1 is overexpressed in many of these diseases, we proposed that VDAC1 could be a common factor mediating metformin’s effects. In this study, we provide direct evidence supporting this hypothesis. We found that metformin induces VDAC1 overexpression and its oligomerization, leading to cell death. Additionally, metformin interacts with hexokinase (HK), causing its detachment from the mitochondria. These VDAC1-related effects, together with metformin activation of the NLRP3 inflammasome, may account for metformin’s diverse effects across various diseases.

## 2. Results

### 2.1. Metformin Induces VDAC1 Overexpression, Oligomerization, and Apoptosis

We have shown that apoptosis inducers induce VDAC1 overexpression, shifting the equilibrium towards oligomers, forming a large channel that enables cytochrome c release, and leading to cell death [5,36,48,50].

**Figure 1:**
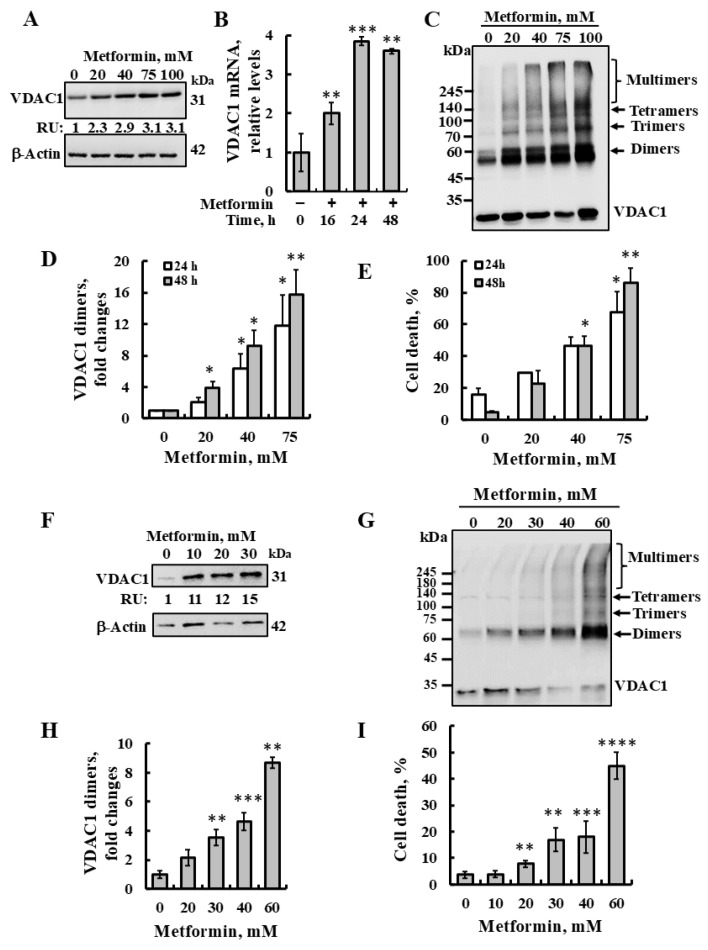
Metformin induces VDAC1 overexpression, oligomerization, and apoptotic cell death. SH-SY5Y (**A**–**E**) or U-87MG (**F**–**I**) cells were incubated with the indicated concentrations of metformin for 24 or 48 h (SH-SY5Y) or 48 h (U-87MG) and subsequently analyzed for VDAC1 expression levels (**A**,**F**) by immunoblotting using anti-VDAC1-specific antibodies. Immunoblotting β-Actin was used as a loading control. VDAC1 levels are presented below the blot in relative units (RUs). (**B**) SH-SY5Y cells were incubated with or without metformin (75 mM), harvested at the indicated times, and subjected to real-time quantitative PCR of VDAC1 mRNA as described in the Materials and Methods section. SH-SY5Y (**C**,**D**) or U-87MG (**G**,**H**) cells were treated as described above and analyzed for VDAC1 oligomerization by incubation protein samples (1 mg/mL) with the cross-linking reagent EGS (100 μM), followed by immunoblotting using anti-VDAC1 antibodies. The positions of VDAC1 monomers, dimers, trimers, tetramers, and higher oligomers are indicated. The level of VDAC1 dimers was analyzed using ImageJ software (version 1.54v) (**D**,**H**) and presented relative to its levels in control cells. Samples were also analyzed for cell death (**E**,**I**) by PI staining and flow cytometry. The results represent the means ± SE; *p* < 0.05 (*), *p* < 0.01 (**), *p* < 0.001 (***), or *p* < 0.0001 (****).

Here, we demonstrated that the incubation of SH-SY5Y cells for 24 or 48 h with metformin resulted, in a concentration-dependent manner, in VDAC1 overexpression at the protein and mRNA levels (Figure 1A,B), oligomerization (Figure 1C,D), and high (80–90%) apoptotic cell death (Figure 1E). This suggests that metformin acts as another apoptosis inducer, enhancing VDAC1 expression which results in its oligomerization, leading to apoptosis. Similar results were obtained using U-87MG cells (Figure 1F–I).

**Figure 2:**
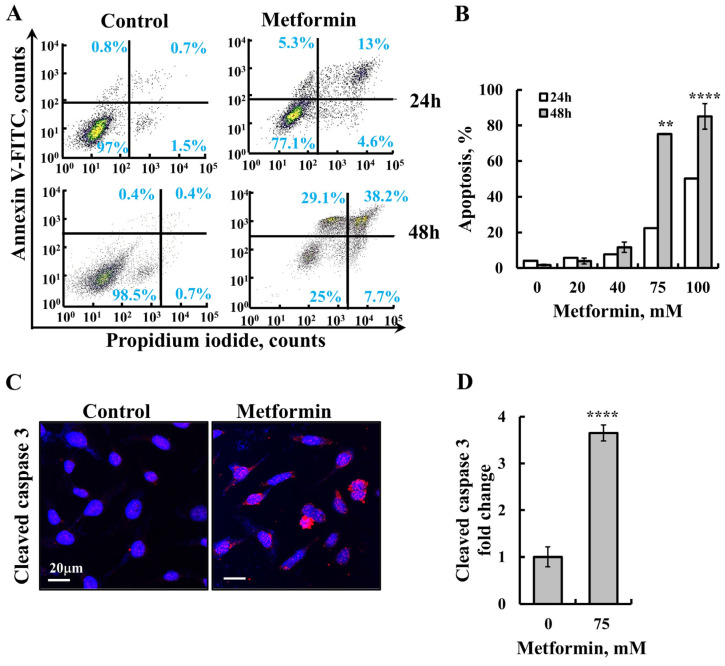
Metformin induces apoptosis. (**A**,**B**) SH-SY5Y cells were treated with the indicated concentrations of metformin for 24 or 48 h, followed by Annexin V–FITC and propidium iodide (PI) staining and flow cytometric analysis to assess apoptotic cell death. (**A**) Representative flow cytometry histograms are shown for control and 75 mM metformin-treated cells at 24 and 48 h. The percentages of live cells, Annexin V-positive cells, Annexin V and PI double-positive cells, and PI-only positive cells are shown. (**B**) Quantification of apoptotic populations from three independent experiments as shown in (**A**). (**C**,**D**) SH-SY5Y cells were cultured on 13 mm glass coverslips, treated with 75 mM metformin for 24 h, fixed, and subjected to IF staining using anti-cleaved caspase-3 antibodies. (**C**) Representative images are shown. (**D**) Quantification of cleaved caspase-3 staining intensity, performed using ImageJ software (version 1.54v). The data represent the mean ± SE from three independent experiments. ** *p* < 0.01; **** *p* < 0.0001.

To more directly assess apoptosis, Annexin V–FITC and propidium iodide (PI) staining were performed followed by flow cytometric analysis, along with the evaluation of cleaved caspase-3 levels (Figure 2). The results demonstrated that metformin induced apoptotic cell death in a concentration-dependent manner (Figure 2A,B). Furthermore, immunofluorescence (IF) staining for activated caspase-3 in SH-SY5Y cells treated with metformin revealed an increase in cleaved caspase-3 levels, further confirming apoptosis induction by metformin (Figure 2C,D).

**Figure 3:**
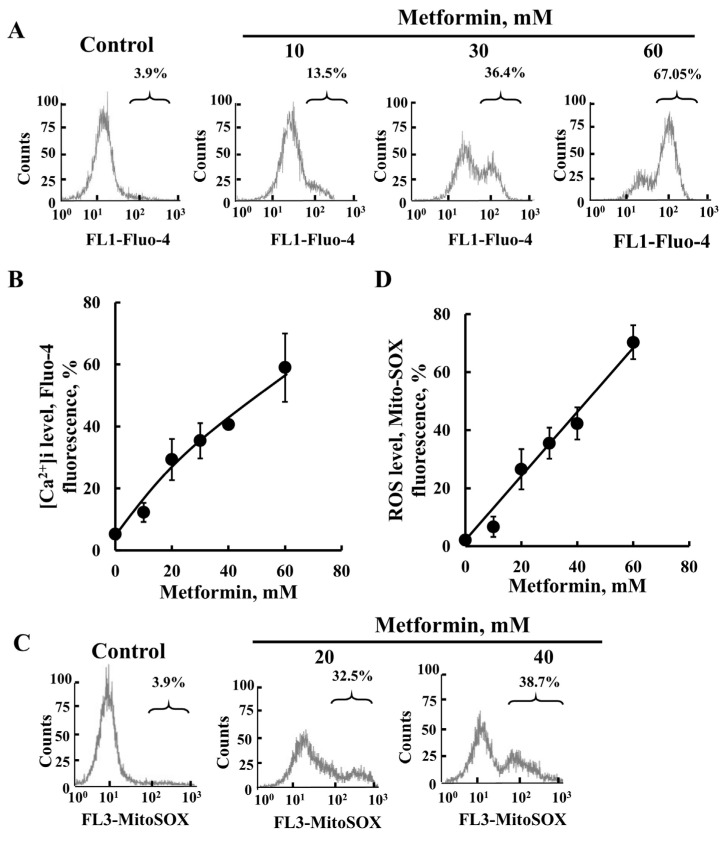
Metformin induces the elevation of cellular [Ca^2+^] and ROS production. SH-SY5Y cells were incubated for 48 h with the indicated concentrations of metformin. Cells were harvested, and the intracellular calcium ([Ca^2+^]i) levels (**A**,**B**) or mitochondrial superoxide levels (**C**,**D**) were measured using Fluo-4-AM or MitoSOX Red^TM^, respectively, and flow cytometry. Representative FACS histograms (**A**,**C**) and quantification (**B**,**D**) are presented. The results represent the means ± SEM (*n* = 3).

Among the changes taking place upon the activation of the intrinsic apoptotic pathway are the increased levels of cytoplasmic [Ca^2+^] and ROS production. Metformin induced an increase in both Ca^2+^ (Figure 3A,B) and ROS production (Figure 3C,D).

The ability of metformin to impair mitochondrial function and induce apoptosis has been previously reported [74]. In MDA-MB-231 cells, metformin at concentrations of 10–40 mM triggered apoptosis, decreased cell viability, ATP production, and mitochondrial membrane potential (∆ψm), while increasing ROS levels. Additionally, it downregulated the anti-apoptotic protein Bcl-2 and upregulated the pro-apoptotic protein Bax. These effects ranged between 40 and 60%, which is notably lower than the up to 90% effect observed in our study. The difference may be attributed to the higher metformin concentrations used in our study (up to 80 mM) and the use of different cell lines [74].

**Figure 4:**
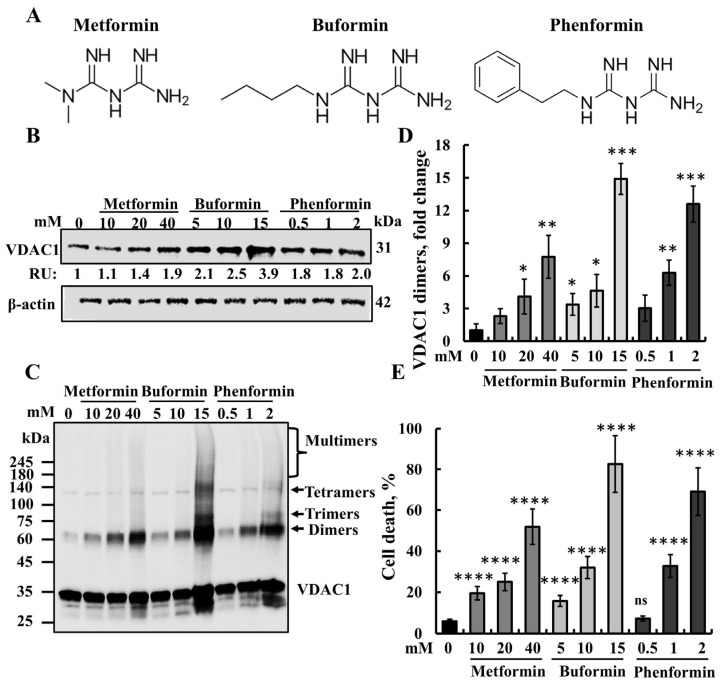
Buformin and phenformin induce VDAC1 overexpression, oligomerization, and apoptotic cell death. (**A**) Chemical structures of metformin and its analogs buformin (1-butylbiguanide) and phenformin. (**B**–**E**) SH-SY5Y cells were incubated for 48 h with the indicated concentrations of metformin, buformin, and phenformin. The cells were analyzed for VDAC1 expression levels (**B**) by immunoblotting using anti-VDAC1-specific antibodies, with β-actin used as a loading control. VDAC1 levels are shown below the blot in relative units (RUs). Additionally, VDAC1 oligomerization was assessed by incubating protein samples (1 mg/mL) with the cross-linking reagent EGS (100 μM), followed by immunoblotting using anti-VDAC1 antibodies. The positions of the VDAC1 monomers, dimers, trimers, tetramers, and higher oligomers are indicated (**C**). The level of VDAC1 dimers was quantified using ImageJ software (**D**) and presented relative to its levels in control cells. Samples were also analyzed for cell death (**E**) by PI staining and flow cytometry. The results represent the means ± SE; *p* < 0.05 (*), *p* < 0.01 (**), *p* < 0.001 (***), or *p* < 0.0001 (****), ns—non-significant.

Next, we asked whether the metformin analogs, buformin and phenformin, induce VDAC1 overexpression, its oligomerization, and apoptosis (Figure 4). Both phenformin and buformin were more potent than metformin in these three effects with cell treatment, with phenformin at 2 mM and buformin at 15 mM producing higher effects than 40 mM of metformin (Figure 4). These different potencies may result from the variance in their cell penetration. Metformin is known to be highly hydrophobic and is transported into the cell by the organic cation transporters (OCTs) [25].

### 2.2. Metformin Binds to the VDAC1 N-Terminal Domain

**Figure 5:**
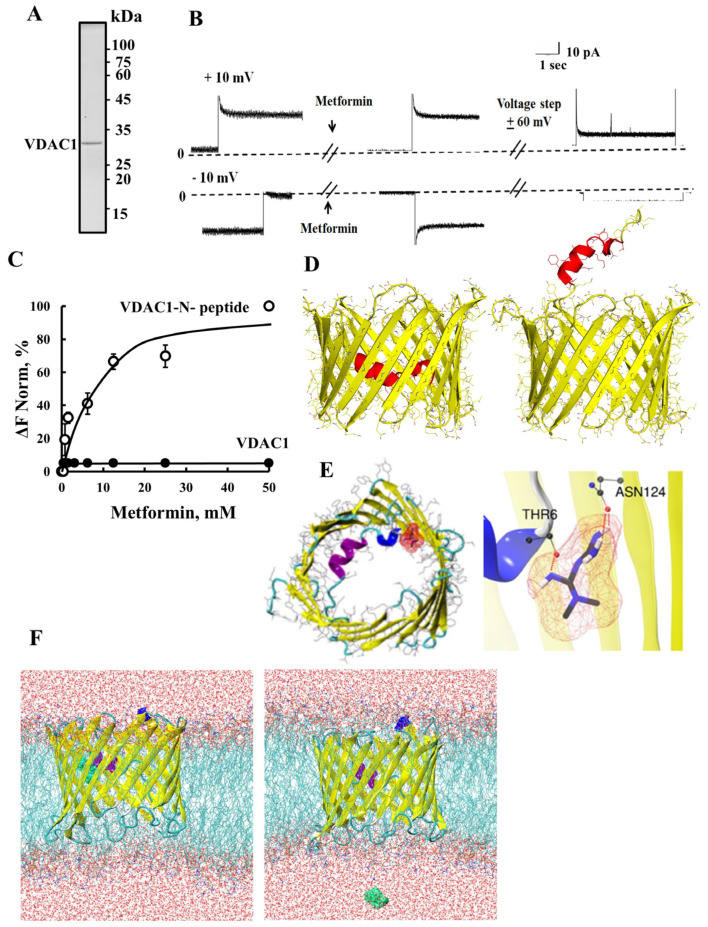
Metformin binds and modulates VDAC1 channel conductance. (**A**) SDS-PAGE of rat liver purified VDAC1 stained with Coomassie blue. (**B**) Purified VDAC1 was reconstituted into a planar lipid bilayer (PLB) prepared from soybean asolectin [43], and single-channel currents through VDAC1, in response to a voltage step from 0 to +10 mV or −10 mV, were recorded before and 5 min after the addition of metformin. Then, the voltage was switched to 60 mV for 1 min and then back to +10 mV or −10 mV, and the current passing through the channels was measured. The dashed lines indicate the zero-level current. (**C**) Fluorescently labeled purified VDAC1 (●; 162 nM) or the FITC-labeled VDAC1-derived *N*-terminus peptide (○; 1–26 amino acids, 2.5 μM) was incubated for 30 min at 37 °C with the indicated concentrations of metformin, and then, micro-scale thermophoresis (MST) was performed. The results are presented as a % of the maximal bound fraction. (**D**) VDAC1 protein with the *N*-terminal domain within and outside of the pore. (**E**) Metformin (in red) docked near the *N*-terminal of the VDAC1, forming hydrogen bonds with threonine 6 and aspargine124 residues. (**F**) The VDAC1-metformin complex equilibrated structure with bilayer of 1-palmitoyl-2-oleoyl-glycero-3-phosphocholine (POPC) and 1-palmitoyl-2-oleoyl-sn-glycero-3-phosphoethanolamine (POPE), with water molecules surrounding the top and bottom of the bilayer. Metformin (green color) during the start of molecular dynamic (MD) simulation, and after 5 ns, showing metformin out of VDAC1.

Next, the direct interaction of metformin with purified VDAC1 (Figure 5A) was assessed using VDAC1 reconstituted into a planar lipid bilayer (PLB) [63] (Figure 5B) and MST (Figure 5C). Metformin reduced VDAC1 conductance only after the channel was exposed to a high voltage step. A similar voltage-dependent effect was found for the binding of HK-I [75] and Bcl-2 to VDAC1 [45]. This effect may result in conformational changes, for example, in the exposure of its *N*-terminus domain. *N*-terminal domain mobility is involved in VDAC1 channel gating [40,44] and in Bax, Bcl-2, and Bcl-xL binding to VDAC1 [43,44,76], as well as in HK binding [75].

As metformin may bind to the *N*-terminus domain, we tested its direct binding to purified VDAC1 protein and to a synthetic peptide representing the VDAC1-*N*-terminus using the MST method (Figure 5C). The results show that metformin does not bind to purified VDAC1 protein, but to its *N*-terminus-derived peptide. This supports the voltage-dependent effect of metformin on channel conductance (Figure 5B).

Next, we performed molecular docking of a neutral state of metformin with VDAC1 protein (PDBID: 3EMN) with the *N*-terminal inside and outside of the pore (Figure 5D) using AutoDock-4.2 docking software [77]. The binding energy of ~4 kcal/mol was observed for metformin with VDAC1 with the *N*-terminal region inside and outside of the channel. The results show that metformin formed three hydrogen bonds with THR6 and ASN124 residues (Figure 5E). In addition, we also explored the docking of different protonation states of metformin, as reported [78]; however, a similar binding energy of ~4 kcal/mol was obtained for metformin with VDAC1 with the *N*-terminal region.

To further validate the interactions of uncharged and protonated metformin with VDAC1, we performed a molecular dynamics (MD) simulation (Figure 5F). To a VDAC1 transmembrane complex, VDAC1 was inserted within the lipid bilayer made up of 1-palmitoyl-2-oleoyl-glycero-3-phosphocholine (POPC) and 1-palmitoyl-2-oleoyl-sn-glycero-3-phosphoethanolamine (POPE) [79]. We used a CHARMM36m forcefield [80] for the system and performed an MD simulation with the GROMACS-[81] simulation package for 50 ns. During the MD trajectory analysis, metformin (uncharged or protonated states) did not bind strongly to the internal cavity of the VDAC1 (*N*-terminal inside or outside of the pore) and came out of the VDAC1 internal cavity at an early stage (~5 ns) in the MD simulation.

These results suggest that metformin binds to the VDAC1 *N*-terminal domain.

### 2.3. Metformin Directly Interacts with Hexokinase and Detaches It from the Mitochondria

Metformin has been shown to inhibit the enzymatic activity of HK-I and HK-II by allosterically modifying their structure, which leads to a reduction in glucose-6-phosphate (G-6-P) production, thereby inhibiting glycolysis [82,83]. Additionally, metformin induces the detachment of HK-II from its binding site in the OMM [82,83].

**Figure 6:**
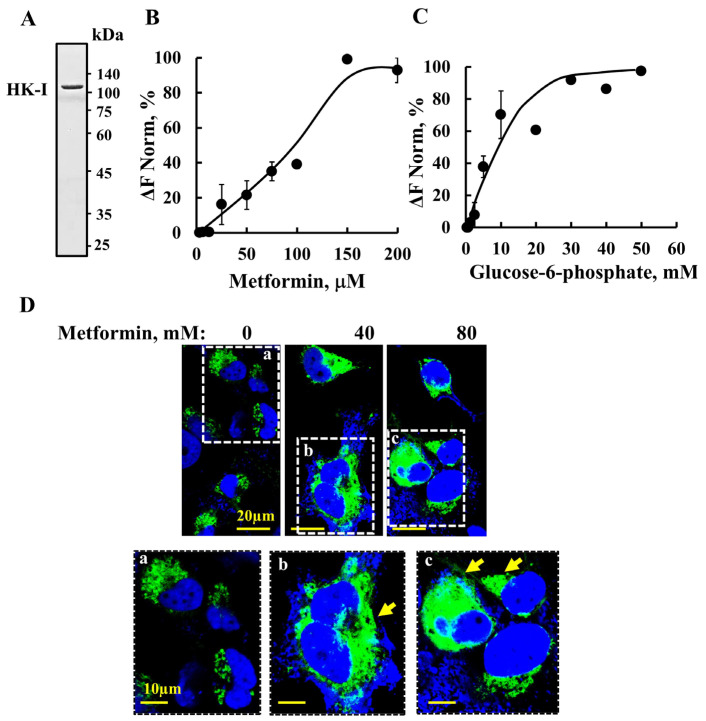
Metformin binds to HK-I and detaches it from the mitochondria. (**A**) Coomassie blue-stained recombinant HK-I was purified as described previously [75]. (**B**,**C**) Fluorescently labeled purified HK-I was incubated for 30 min at 37 °C with increasing concentrations of metformin (5 to 200 μM) (**B**) or with glucose-6-phosphate (0.6–60 mM) (**C**), and then micro-scale thermophoresis (MST) was performed. The results are presented as a % of the bound fraction. (**D**) SH-SY5Y cells were seeded on 13 mm glass coverslips, transfected to express HK-I-GFP (24 h, 1 μg, green staining), and incubated for 4 h with and without the indicated concentration of metformin. They were fixed, stained with DAPI (blue staining), and visualized under confocal microscopy. Enlarged areas in dashed squares are indicated as (**a**–**c**), with the arrows pointing to diffused HK-GFP. The results represent the means ± SEM (n = 3).

Here, we have shown that metformin binds to purified HK-I (Figure 6A) in a concentration-dependent manner, as revealed using MST methods (Figure 6B). Interestingly, although metformin induces VDAC1 overexpression and cell death at high metformin concentrations (20–75 mM) (Figure 1 and Figure 2), the half-maximal binding (C_50_) of metformin to HK-I was obtained at 100 μM, which are 200- to 750-fold lower concentrations.

For comparison, the binding of G-6-P to HK-I was also analyzed, showing binding with C_50_ of 10 mM (Figure 6C). The binding of G-6-P to HK inhibits its activity at over 200-fold higher concentrations than those of metformin.

To monitor the metformin detachment of HK-I from the mitochondria/VDAC1, cells were transfected to express HK-I-GFP and incubated with and without metformin. In the absence of metformin, HK-I-GFP florescence is punctuated, suggesting that it is bound to the mitochondria (Figure 6Da). In the presence of metformin, HK-I-GFP fluorescence is diffused in the cell, suggesting its detachment from mitochondria/VDAC1 (Figure 6Db,c). This suggests that metformin, by binding to HK-I, induces conformational changes in HK-I, leading to its detachment from VDAC1.

### 2.4. Metformin Increased NLRP3 and ASC Levels

**Figure 7:**
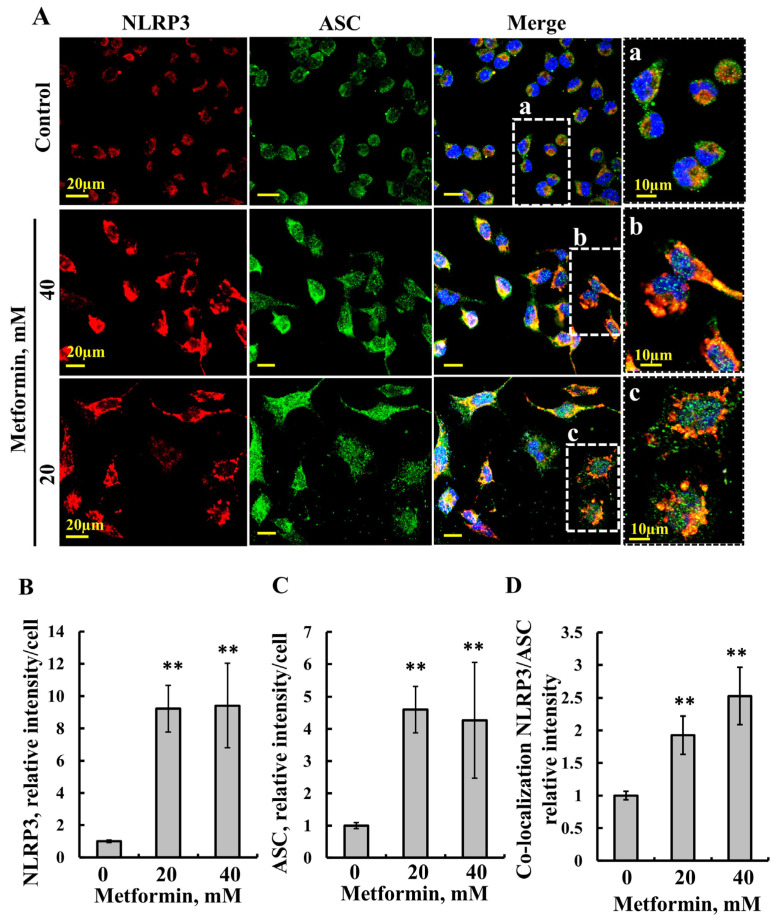
Metformin induces the activation of the NLRP3 inflammasome. SH-SY5Y cells were seeded on 13 mm glass coverslips and then incubated for 48 h with the indicated concentrations of metformin, followed by co-immuno-staining with anti-NLRP3 (red staining) and anti-ASC (green staining) antibodies. Cells then were stained with DAPI (blue staining) and images were visualized by confocal microscopy (**A**), and the staining intensity of NLRP3 (**B**), ASC (**C**), and their co-localization (**D**), as analyzed using Image J software (version 1.54v) is presented. The results represent the means ± SE; *p* < 0.01 (**).

The nucleotide-binding and oligomerization domain (NOD)-like receptors (NLRs) are pattern-recognition receptors, with the family member known as the NLR pyrin domain containing three (NLRP3) forms the inflammasome. The activation of NLRP3 inflammasome is associated with mitochondrial dysfunction, increased ROS production, and the release of mitochondrial DNA (mtDNA) into the cytosol to evoke an inflammatory response [84,85]. As metformin inducing VDAC1 oligomerization was found to mediate the release of mtDNA, we tested its effect on the NLRP3 inflammasome (Figure 7). Metformin highly increased the expression of NLRP3 and that of the apoptosis-associated speck-like protein containing a CARD (ASC), leading to NLRP3 inflammasome assembly and activation [86]. The results show that metformin treatment increased the expression of NLRP3 (Figure 7A,B) and ASC (Figure 7A,C), and it induced their co-localization (Figure 7A,D) and the formation of large intracellular assemblies.

## 3. Discussion

Metformin, a well-established anti-diabetic medication, is gaining attention for its potential re-purpose in cancer treatment, yet its efficacy in cancer therapy remains variable [87,88]. This re-purposing trend, encapsulated in the concept of “Metformin, the aspirin of the 21st century” [89], underscores its relevance in the realms of cancer and aging [90]. An accepted view is that metformin exerts anti-tumor effects through various proposed mechanisms, including the modulation of the cellular AMP/ATP ratio, activation of the AMPK/mTOR pathway, inhibition of mitochondrial complex I, and suppression of gluconeogenesis [15,16]. However, some of these proposed mechanisms have been challenged [91].

In this study, we demonstrate that VDAC1 is a target for metformin and its analogs.

### 3.1. Metformin-Induced Apoptosis Can Be Mediated via VDAC1

**Figure 8:**
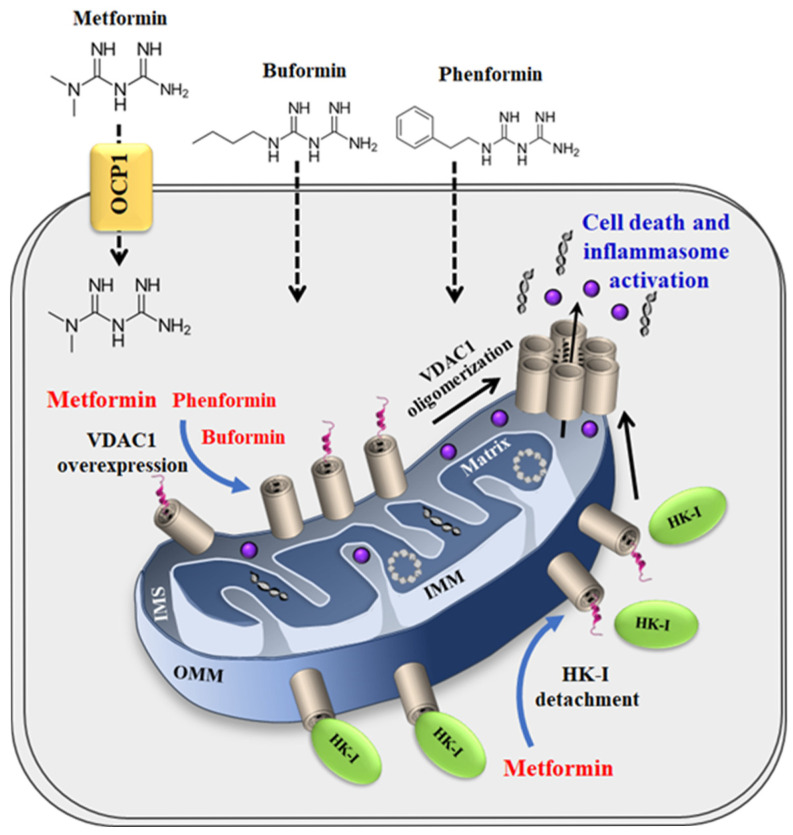
Proposed model for metformin inducing VDAC1 overexpression, oligomerization, and apoptosis. Metformin entering the cell by the transporter OCP1 and hydrophobic buformin and phenformin directly crossing the cell membrane all enhancing VDAC1 expression levels with the overexpression of VDAC1 shifting the equilibrium towards the VDAC1 oligomeric state. This mediates the release of apoptogenic proteins and mitochondrial DNA (mtDNA), leading to apoptosis and inflammation. Metformin detaches HK-I from VDAC1, further enhancing VDAC1 oligomerization, which results in apoptosis and inflammation.

Apoptosis inducers, stress, and pathological conditions have been shown to induce VDAC1 overexpression and its oligomerization, thus triggering apoptosis [36,51]. The results presented here suggest a similar mechanism for metformin (Figure 8). At relatively high concentrations, it induces the overexpression and oligomerization of VDAC1, leading to cell death (Figure 1 and Figure 2). Similar effects were obtained with the metformin analogs, buformin and phenformin (Figure 4), which further support VDAC1 as a target of metformin. Like metformin, they induce VDAC1 overexpression, its oligomerization, and cell death. These effects were obtained at lower concentrations than with metformin and showed the same concentration dependence, suggesting a link between them by buformin and phenformin (Figure 4).

Considering the pro-apoptotic effects associated with VDAC1 overexpression, we propose that metformin, as other apoptosis inducers and stress conditions [36,51], triggers apoptosis by inducing VDAC1 overexpression. Intriguingly, it also impacts the expression of other apoptosis-associated proteins, upregulating the expression of p53, Bax, and Bad, while concurrently downregulating the expression levels of anti-apoptotic proteins such as Bcl-2 [74,92].

An increase in VDAC1 expression was observed in the cortex, but not in the hippocampus of mice treated with metformin for three months [13]. Furthermore, in the cortical region, oligomeric plasmalemmal VDAC1 (pl-VDAC1) was detected in areas where metformin triggered Aβ-aggregate accumulation and apoptosis in neurons [13]. Additionally, metformin elevated VDAC1 expression levels in polycystic ovary syndrome-like rats [93].

Pro-apoptotic agents were shown to induce the upregulation of VDAC1 expression levels in a Ca^2+^-dependent manner [51]. Here, we show that metformin increases both [Ca^2+^]i and ROS levels (Figure 3). Metformin has been reported to induce ER stress and subsequent Ca^2+^ released from the ER, thereby it is increased in the cytosol [94]. Consequently, the elevation in [Ca^2+^]i may contribute to VDAC1 overexpression, as found for other inducers of VDAC1 overexpression [51].

Recently, we observed that VDAC1 expression levels are elevated in islets from both T2DM and non-diabetic organ donors under glucotoxic conditions [63]. High glucose concentrations further increase VDAC1 expression levels by upregulating the transcription factors SREBP1 and SREBP2, which regulate VDAC1 expression [95]. Consequently, the overexpressed VDAC1 is mistargeted to the plasma membrane of the insulin-secreting β cells, leading to ATP loss and the subsequent impairment of insulin secretion [63,95]. Moreover, VDAC1 antibodies, as well as metformin, and the specific VDAC1-interacting molecule VBIT-4 that inhibits its oligomerization restored impaired ATP generation and glucose-stimulated insulin secretion in T2DM islets and in db/db mice, maintaining normal glucose tolerance and restored insulin secretion [63].

Metformin not only induces VDAC1 overexpression, but it also directly interacts with VDAC1, as evidenced by its inhibition of the channel conductance of bilayer-reconstituted VDAC1 (Figure 5) [63]. We identified the VDAC1 *N*-terminus as the metformin-interacting site, as demonstrated by its binding to the *N*-terminal-derived peptide (Figure 5C), and an in silico analysis of the metformin binding site in VDAC1 revealed its interaction with the *N*-terminus threonine 6 (Figure 5E). Intestinally, computational alanine scanning mutagenesis revealed that metformin binding to VDAC1 involves amino acid aspartate 9, which is involved in electrostatic interactions with metformin [94]. This interaction likely modulates VDAC1 activity, subsequently influencing mitochondrial functions. Therefore, metformin’s impact on metabolism may be attributed to its interaction with VDAC1.

These findings, as summarized in Figure 8, support the currently well-established concept of drugs, stress, and pathological conditions leading to cell death via the induction of VDAC1 overexpression, which results in its oligomerization, forming a mega channel that allows the release of pro-apoptotic and mitochondrial DNA, culminating in apoptosis and inflammation [48,50,51,52].

It should be noted, however, that other proposed mechanisms of metformin-induced cell death are also active. Reducing VDAC1 expression using specific siRNA only slightly reduced metformin-induced cell death. Similar results were obtained with DIDS (4,4′-Diisothiocyanatostilbene-2,2′-disulfonic acid), a known VDAC1 inhibitor [52]. These findings suggest that in the absence of VDAC1, or when its oligomerization is inhibited [52], alternative metformin-mediated mechanisms of cell death may become the predominant pathways.

### 3.2. Metformin Interaction with HK and Its Detachment from VDAC1

Cancer cells express high levels of mitochondria-bound HK [96], which catalyzes the first step of glycolysis. HK binds to VDAC1 [34,41,42,43], while the VDAC1-HK complex allows the utilization of newly synthesized ATP to phosphorylate glucose, thereby establishing a coupling between the glycolytic pathway and oxidative phosphorylation (OXPHOS). This regulates cellular metabolism by governing the glycolytic pathway, which supplies metabolic intermediates that are crucial for cancer cell survival. HK’s interaction with VDAC1 also protects against mitochondria-mediated apoptosis [41,42,44,75,97].

Hence, HK binding to VDAC1 confers both metabolic advantages and the suppression of apoptosis. Consequently, the HK-VDAC1 complex has emerged as a target for anti-cancer drugs [98]. Indeed, various compounds have been developed to disrupt the association between HK and VDAC1. These induce peptides derived from HK-I [99], methyl-jasmonate [100], and VDAC1-based peptides [41,44,75].

Metformin has been shown to modulate the glycolytic rate by directly inhibiting HK-II activity [101] and its association with the mitochondria [83]. In silico models suggest that metformin mimics the features of G-6-P and binds to its binding site in HK [82]. In this study, we demonstrated that, similar to G-6-P, metformin directly binds to purified HK-I with the relatively high affinity of 100 µM, which is 150-fold higher than that of G-6-P showing C_50_ of 15 mM (Figure 6). The binding of G-6-P to HK-I at high concentrations is in accord with product inhibition when it accumulates. Moreover, G-6-P plays an essential role in glycogen synthesis and the pentose phosphate pathway. Therefore, by binding to the G-6-P binding site in HK [82], metformin not only affects glycolysis, but it also influences glycogen synthesis and the pentose phosphate pathway.

Metformin, by binding to HK, serves a dual role as an HK inhibitor [101] and in VDAC detachment (Figure 6). It is important to note that both HK-I and metformin interact with the VDAC1-*N*-terminuse (Figure 5). Consequently, the overexpression of HK-I and HK-II in most cancer cells ensures an energy supply and protection against mitochondria-mediated apoptosis [41,42,44,75,97]. Thus, metformin, by inhibiting HK activity and inducing its detachment from VDAC1, results in both inhibiting cancer cell metabolism and inducing apoptosis.

### 3.3. Metformin and Inflammation

Mitochondria have been implicated in inflammation through the regulation of the NLRP3 inflammasome and nucleation [84,102,103,104] and in immunity [105]. VDAC1 has been implicated in inflammasome activation [102,106] and is essential for the activation of the NRLP3 inflammasome [53,70,107,108,109]. Additionally, VDAC1 oligomers have been shown to act as a docking site for NRLP3 during the initial assembly of the multi-protein oligomeric NLRP3 inflammasome complex [55]. In this study, we demonstrated that metformin significantly upregulated the expression of NLRP3 and ASC, key components involved in NLRP3 inflammasome assembly and activation [86]. Furthermore, metformin promoted their co-localization and the formation of large intracellular assemblies. These structures may represent filament formation, consistent with previous findings showing that activated NLRP3 drives ASC polymerization into filamentous structures [110,111,112].

VDAC1 can oligomerize in response to various signals, allowing the release of pro-apoptotic proteins and mtDNA from the mitochondria [53], thereby triggering apoptosis and inflammation. As metformin induces increased ROS and [Ca^2+^] levels and induces VDAC1 oligomerization, the increase seen in NLRP3 levels and assembly may be related to these effects. This is supported by the findings that the VDAC1 oligomerization inhibitor, VBIT-4, inhibits NLRP3 inflammasome activation [55,107], suggesting that VDAC1 oligomerization is a critical step in inflammation.

Moreover, it has been demonstrated that the dissociation of HK from VDAC1 triggers the assembly and activation of the NLRP3 inflammasome, with the dissociation of HK-II from VDAC1 being a common step in NLRP3 inflammasome activation [55]. Thus, metformin, by inducing HK detachment from VDAC1, promotes VDAC oligomerization and therefore apoptosis and inflammation.

### 3.4. Metformin, Buformin, and Phenformin as Potential Cancer Treatments

Metformin, buformin, and phenformin are all biguanides, a class of drugs traditionally used to treat type 2 diabetes and lower blood glucose levels. However, their potential roles in cancer treatment have gained increasing attention in recent years due to their effects on cellular metabolism and signaling pathways that are also implicated in cancer development and progression.

The anti-cancer activity of metformin involves complex molecular mechanisms, targeting multiple pathways. These mechanisms include the activation of the LKB1/AMPK pathway, which leads to AMPK activation and the inhibition of mTOR, MEK/ERK, and PI3K/AKT signaling. Additionally, metformin demonstrates a potent anti-proliferative effect on cancer cell lines derived from the breast, colon, ovaries, pancreas, lung, and prostate, as well as pancreatic, colorectal, and leukemia cancers [113,114,115,116]. Metformin-induced apoptosis inhibiting mitochondria creates an energy crisis within cancer stem cells [92,117]. Furthermore, metformin activates the immune system and eradicates cancer stem cells within tumors, enhancing the responsiveness of glioma cells to temozolomide [118,119]. However, it is important to note that some clinical trials have failed to demonstrate a protective association between metformin and survival in colorectal cancer patients with T2DM [120,121]. Clinical trials are ongoing to determine its efficacy as an adjunct therapy for cancer patients, particularly in combination with other cancer treatments [92,117].

Phenformin was the most potent of the biguanides but was withdrawn from clinical use due to its high risk of causing lactic acidosis. Like metformin, phenformin can activate AMPK, inhibit mTOR signaling, and suppress cellular growth and proliferation [29]. Additional clinical studies recently recommended re-purposing phenformin as an anti-tumor drug [122]. Phenformin has also been shown to exhibit synergistic effects when combined with other anti-cancer agents in reducing tumor growth, metastatic potential, and cancer stem cells, while inducing apoptosis [123].

Similarly to phenformin, buformin’s potential as an anti-cancer agent arises from its ability to disrupt cancer cell metabolism by inhibiting mitochondrial complex I, activating AMPK, inducing oxidative stress, and promoting autophagy. However, the risk of side effects such as lactic acidosis and the need for further clinical evaluation remain important factors to consider for its use in cancer therapy. Buformin also exhibits anti-proliferative and anti-invasive effects in the cells of endometrial [124], lung [125], and cervical cancers [126]. In combination with 2-deoxy-glucose or WZB-117, it inhibits the viability of highly resistant human lung cancer cells [125].

In conclusion, in this study, we proposed VDAC1 as a target for metformin. We demonstrated that metformin and its analogs, phenformin and buformin, induce VDAC1 overexpression, promote its oligomerization, and subsequently, trigger apoptotic cell death. Furthermore, metformin binds to HK, detaching it from VDAC1, which not only disrupts energy production, but it also facilitates VDAC1 oligomerization and apoptosis.

However, the precise binding site of metformin on VDAC1, as well as the specific mechanisms or transcription factors responsible for metformin-induced VDAC1 overexpression, remain to be elucidated.

Given the multi-functionality of VDAC1, its interaction with metformin may elucidate why the drug does not conform to the paradigm of the “one molecule–one target–one disease” model but rather represents a “magic bullet” approach.

## 4. Materials and Methods

### 4.1. Materials

Metformin (Cat. No: 317240), buformin (Cat. No: SML1496), henformin (Cat. No: PHR1573), propidium-iodide (PI), 4′,6-diamidino-2-phenylindole (DAPI), Ponceau S, tris(hydroxymethyl)aminomethane (TRIS), Tween-20, trizol, and protease inhibitor cocktail were obtained from Sigma (St. Louis, MO, USA). Phosphate-buffer saline (PBS), Dulbecco’s modified Eagle’s medium (DMEM) media, the supplement fetal bovine serum (FBS), and penicillin-streptomycin were obtained from Gibco (Grand Island, NY, USA). Paraformaldehyde and formaldehyde were obtained from Emsdiasum (Hatfield, PA, USA). A chemiluminescence detection kit was from Advansta Inc. (San Jose, CA, USA). Polyvalent JetPrime transfection reagent was from Polyplus (llkirch-Graffenstaden, France). Fast SYBR™ Green Master Mix, Ethylene glycolbis (succinimidyl succinate) (EGS), MitoSOX^TM^ red mitochondrial superoxide indicator, and Fluo-4 AM were obtained from Thermo Fisher Scientific (Waltham, MA, USA). All-in-one 5X RT Master mix was obtained from Applied Biological Materials (Richmond, BC, Canada). Hank’s Balanced Salt Solution (HBSS) was from Biological Industries (Beit-Haemek, Israel). The primary and (HRP)-conjugated and fluorophore-conjugated secondary antibodies, their sources, and the dilutions used are detailed in Table 1.

### 4.2. Cell Culture and Metformin, Buformin, or Phenformin Treatment

SHSY5Y (human neuroblastoma) and U-87MG (human glioblastoma) cell lines were obtained from the American Type Culture Collection (ATCC) (Manassas, VA, USA) and were maintained as per the ATCC instructions. Cells were maintained in DMEM growth medium supplemented with 10% heat-inactivated fetal bovine serum (FBS), 100 U/mL penicillin, and 100 μg/mL streptomycin. Cells were grown at 37 °C in 5% CO_2_ in a humidified incubator. Cell lines were routinely tested for mycoplasma contamination.

Cells at 60% confluence were incubated with various concentrations of metformin, buformin, or phenformin for 24 h or 48 h. Then, cells were trypsinized, centrifuged (1500× *g*, 5 min), washed with PBS, and subjected to the desired assay.

### 4.3. HK-I Detachment

Cells were grown on coverslips in a 12-well plate and were transfected with pEGFP-HK-I, encoding an HK-I-GFP fusion protein, where GFP was connected to the C-termimus of HK-I, as described previously [41]. Logarithmically growing SH-SY5Y cells were transiently transfected with HK-I-GFP (1 μg) using JetPRIME transfection reagent (Polyplus, llkirch-Graffenstaden, France) according to the manufacturer’s instructions. After 24 h, cells were treated with metformin for an additional 24 h. Cells were then washed with PBS and fixed with 4% paraformaldehyde for 15 min at room temperature. Then, cells were stained with DAPI, mounted with Fluoroshield mounting medium (Immunobioscience, Mukilteo, WA) and visualized using a confocal microscope (Olympus IX81).

### 4.4. VDAC1 Oligomerization Assay

Cells were treated with metformin, buformin, or phenformin as indicated above, harvested, and washed with PBS, at pH 8.3, and then incubated at 1 mg/mL with or without EGS (100 μM, 15 min, 30 °C). Samples were analyzed for VDAC1 oligomerization (30–40 µg protein) and subjected to SDS-PAGE and immunoblotting using anti-VDAC1 antibodies. The immuno-reactive bands corresponding to VDAC1 monomers, dimers, and multimers were analyzed using a FUSION-FX system (Vilber Lourmat, Collégien, France) and ImageJ software.

### 4.5. Protein Extraction from Cells, Gel Electrophoresis, and Immunoblotting

Cells were subjected to the desired treatment and lysed using lysis buffer (50 mM Tris-HCl, pH 7.5, 150 mM NaCl, 1 mM EDTA, 1.5 mM MgCl_2_, 10% glycerol, 1% Triton X-100), supplemented with a protease inhibitor cocktail (Calbiochem, San Diego, CA, USA). The lysates were then vortexed and heated at 70 °C for 10 min. Finally, cell lysates were centrifuged (15,000× *g*, 10 min at 4 °C), and the protein concentration of the supernatant was determined. Protein samples were stored at −80 °C until further use in gel electrophoresis.

Protein aliquots (10–20 μg) were subjected to SDS-PAGE and were then electro-transferred onto nitrocellulose membranes for immunostaining. The membranes were incubated with a blocking solution containing 5% non-fat dry milk and 0.1% Tween-20 in tris-buffered saline (TBST), followed by incubation with primary antibodies (Table 1). Subsequently, membranes were incubated with HRP-conjugated anti-mouse or anti-rabbit IgG as secondary antibodies. Band intensities were visualized using FUSION-FX (Vilber Lourmat) and quantified using ImageJ (version 1.54v; Sun Microsystems, Santa Clara, CA, USA) software, and the values were normalized to the intensities of the appropriate β-actin signal that served as a loading control.

### 4.6. Cell Death

Cell death was analyzed using propidium iodide (PI, 6.25 μg/mL) staining, performed according to the recommended protocol, followed by flow cytometry using an iCyt sy3200 Benchtop Cell Sorter/Analyzer and EC800 software (Sony Biotechnology Inc., San Jose, CA, USA). Cell death was also evaluated using PI and annexin V-fluorescein isothiocyanate (FITC) (Annexin V–FITC) according to the recommended protocol. Cells were incubated for 15 min with the reagents, protected from light, then washed once with the binding buffer (10 mM HEPES/NaOH, pH 7.4, 140 mM NaCl, and 2.5 mM CaCl_2_), resuspended in 200 μL binding buffer, and analyzed by flow cytometry. At least 10,000 events were collected, recorded on a dot plot, and analyzed using ec800 flow cytometer software (Sony Biotechnology Inc., San Jose, CA, USA).

### 4.7. Real-Time Quantitative PCR (q-RT-PCR) Analysis

Total RNA was isolated from SH-SY5Y cells using the trisol reagent method. VDAC1 mRNA quantification was carried out using the following primers: forward 5′-AATGACGGGACAGAGTTTGGCCA-3′ and reverse 5′-AGCGCGTGTTACTGTTTCCTGCCA-3′, synthesized by HyLabs (Rehovot, Israel). Real-time quantitative PCR was carried out using AB 7300 Sequence Detection Software (Applied Biosystems; Waltham, MA, USA), using SYBR Green master mix reagent Thermo Fisher Scientific (Waltham, MA, USA) according to the manufacturer’s instructions. The threshold cycle (Ct) was defined as the number of cycles required for the fluorescence intensity to rise above the background fluorescence. A standard curve was generated for each set of primers using different amounts of cDNA and plotting Ct as a function of the amount of total cDNA added to the reaction. Relative expression levels for each gene in each sample were calculated by the ddCT-based calibrated standard curve method. The mean fold changes (±SEM) of the three replicates were calculated.

### 4.8. Micro-Scale Thermophoresis (MST)

An MST analysis was conducted using a NanoTemper Monolith NT.115 apparatus, as described previously [127]. Briefly, purified VDAC1, VDAC1-*N*-terminal (1–26 amino acids) peptide, and HK-I were fluorescently labeled using a NanoTemper Protein labeling kit BLUE (L001, NanoTemper Technologies, München, Germany). A constant concentration of VDAC1 (162 nM), HK-I (112.5 nM), or VDAC1-*N*-terminal peptide (2.5 μM) was incubated in 10 mM Tricine-buffer, at pH 7.4, with the varying concentrations of metformin or glucose-6-phosphate, as indicated in the legend of Figure 6. After a 30 min incubation, 8 µL of each sample was loaded into glass capillaries (Monolith NT Capillaries from NanoTemper Technologies, München, Germany)), and thermophoresis was analyzed.

### 4.9. Intracellular Ca^2+^ Level Analysis

Fluo-4-AM (Thermo Fisher; Waltham, MA, USA) was used to monitor changes in cytosolic Ca^2+^ levels. Cells were harvested, collected (1500× *g* for 5 min), washed with Hank’s Balanced Salt Solution (HBSS) supplemented with 1.8 mM CaCl_2_ (HBSS+), and incubated with 2 μM Fluo-4 in 200 μL HBSS(+) buffer for 30 min at 37 °C in a light-protected environment. After removing excess dye by washing with HBSS(+), the cellular free Ca^2+^ concentration was promptly measured using an iCyt sy3200 Benchtop Cell Sorter/Analyzer (Sony Biotechnology Inc., San Jose, CA, USA). At least 10,000 events were recorded by the FL1 detector, represented as a histogram, and analyzed using ec800 software (Sony Biotechnology Inc., San Jose, CA, USA). Positive cells exhibited a shift to an enhanced level of green fluorescence (FL1).

### 4.10. Reactive Oxygen Species (ROS) Level Analysis

To assess mitochondrial ROS accumulation, cells were collected and then treated with MitoSOX™ Red (Thermo Fisher; Waltham, MA, USA), a mitochondrial superoxide indicator for live-cell imaging, for 10 min at 37 °C. At least 10,000 events were recorded by the FL2 detector, represented as a histogram, and analyzed by EC800 software (Sony Biotechnology Inc., San Jose, CA, USA).

### 4.11. HK-I and VDAC1 Purification and Channel Activity Reconstitution into a Planar Lipid Bilayer (PLB)

VDAC1 was purified from rat liver mitochondria, reconstituted into a PLB, and single- and multiple-channel current recording with data analysis was carried out, as described previously [43]. Briefly, a PLB was prepared from soybean asolectin, and VDAC1 was added to one side of the PLB. After one or a few channels were inserted into it, the excess protein was removed by perfusing and passing currents through the VDAC1, in response to a voltage step from 0 to +10 mV or −10 mV, that was recorded before and 5 min after the addition of metformin. As indicated, in some recordings, the voltage was switched to 60 mV for 1 min and then back to +10 mV or −10 mV, and the current passing through the channels was measured under a voltage clamp using a BC-535B bilayer clamp amplifier (Warner Instruments, Hamden, CT, USA).

### 4.12. Immunofluorescence (IF) Staining

For IF staining, cells were grown on coverslips and subjected to the desired treatment. Cells were then washed with PBS and fixed with 4% paraformaldehyde (15 min, RT). The cells were washed with PBS, then incubated with blocking buffer (10% NGS, 1% BSA, and 0.1% Triton X-100) for 2 h and washed with PBST (PBS containing 0.1% Triton X-100). They were then incubated with the primary antibodies overnight at 4 °C. Afterwards, the cells were washed and incubated in the dark with secondary antibodies (2 h, RT), stained with DAPI (0.5 μg/mL) (nucleus), washed, mounted with Fluoroshield mounting medium (Immunobioscience, Mukilteo, WA, USA), and imaged by confocal microscopy (Olympus 1X81, Shinjuku, Tokyo, Japan).

### 4.13. Statistical Analysis

The data are presented as the mean ± SEM of at least three independent experiments, unless otherwise specified. Statistical significance was determined using a two-tailed Student’s *t*-test in Microsoft Excel. A *p*-value of ≤0.05 (*), ≤0.01 (**), ≤0.001 (***), or ≤0.0001 (****) was considered statistically significant.

## Figures and Tables

**Table 1:** Antibodies used in this study. Antibodies against the indicated protein, their catalog number, source, and the dilutions used in immunoblotting (WB) and immunofluorescence (IF) experiments.

Antibody	Source and Catalog Number	Dilution	
		WB	IF
Mouse monoclonal anti-β-Actin	Millipore, Billerica, MA, USA, MAB1501	1:40,000	-
Goat anti-rabbit—HRP	Promega, Madison, WI, USA, W4011	1:15,000	-
Goat anti-rabbit—Alexa Fluor 555	Abcam, Cambridge, UK, ab150078	-	1:750
Goat anti-mouse H and L—Alexa Fluor 488	Abcam, Cambridge, UK, ab150114	-	1:750
Rabbit polyclonal anti-VDAC1	Abcam, Cambridge, UK, ab15895	1:5000	1:500
Mouse monoclonal anti-ASC	Santa Cruz Biotechnology, Santa Cruz, TX, USA, sc-514414		1:500
Rabbit polyclonal anti-NLRP3	Novus Biologicals, Centennial, CO, USA, NBP1-77080		1:500
Rabbit polyclonal anti cleaved caspase-3	Cell signaling, Danvers, MS, USA, 9661S		1:400

## Data Availability

The authors agree that all data and materials generated from this study will be shared with other qualified investigators through academically established means.

## Abbreviations

The following abbreviations are used in this manuscript:

AMPK	AMP-activated kinase
AD	Alzheimer’s disease
ASC	Apoptosis-associated speck-like protein containing a CARD
CVDs	Cardiovascular diseases
DPC	Diphenylamine-2-carboxylate
G-6-P	Glucose-6-phosphate
HK	Hexokinase
IBDs	Inflammatory bowel diseases
mTOR	Mammalian target of rapamycin
mtDNA	Mitochondria DNA
MST	Micro-scale thermophoresis
NAFLD	Non-alcoholic fatty liver disease
NLRP3	NLR pyrin domain contains three
OCTs	Organic cation transporters
PLB	Planar lipid bilayer
ROS	Reactive oxygen species
T2DM	Type 2 diabetes mellitus
VDAC1	Voltage-dependent anion channel
